# Assessment of the effectiveness of psychosocial rehabilitation in schizophrenia patients using biological markers

**DOI:** 10.1192/j.eurpsy.2021.1350

**Published:** 2021-08-13

**Authors:** S. Zozulya, V. Yastrebova, T. Solokhina, T. Klyushnik

**Affiliations:** 1 Laboratory Of Neuroimmunology, Mental Health Research Centre, Moscow, Russian Federation; 2 Department Of Mental Health Support Systems Research Centre, Mental Health Research Centre, Moscow, Russian Federation

**Keywords:** schizophrénia, Psychosocial rehabilitation, inflammatory and autoimmune markers

## Abstract

**Introduction:**

An important aspect of rehabilitation programmes is assessment of their effectiveness, which is carried out mainly through clinical and psycho-pathological examinations, psychometric and psychological scales and questionnaires. The use of biological markers of the schizophrenic process to assess the effectiveness of rehabilitation assistance is of considerable interest.

**Objectives:**

To compare the clinical and socio-psychological characteristics of schizophrenia patients receiving psychosocial treatment in various forms of psychiatric care with the level of immune system activation reflecting the activity and severity of the pathological process in brain.

**Methods:**

77 schizophrenia patients in remission of varying quality were examined, of which 52 patients (the 1^st^ group) participated in a long-term comprehensive rehabilitation programme (3.7±2.5 years) in non-profit organization, and 25 patients (the 2^nd^ group) received medical and rehabilitation assistance in the psychiatric day hospital (duration of treatment no more than 60 days). PANSS, HDRS, SAS-SR, SF-36, BRS scales were used. The activity of the pathological process was evaluated by the level of inflammatory markers.

**Results:**

Both patient groups showed a similar increase in the level of inflammatory and autoimmune markers compared to control (p<0,01). The 1^st^ group compared to the 2^nd^ one had a significantly higher level (p<0,05) of social functioning, stress resistance, awareness of the disease, motivation, comprehensiveness, as well as less the severity of psychopathological symptoms.

**Conclusions:**

The results indicate the effectiveness of a long-term comprehensive rehabilitation programme to stabilize clinical remission, improve social functioning and the quality of life in schizophrenia patients, despite the active pathological process in brain.

